# Super Time‐Resolved Tomography

**DOI:** 10.1002/advs.202511933

**Published:** 2025-10-30

**Authors:** Zhe Hu, Zisheng Yao, Kalle Josefsson, Francisco García‐Moreno, Malgorzata Makowska, Yuhe Zhang, Pablo Villanueva‐Perez

**Affiliations:** ^1^ Synchrotron Radiation Research and NanoLund Lund University Lund 22100 Sweden; ^2^ Institute of Applied Materials Helmholtz‐Zentrum Berlin 20036 Berlin Germany; ^3^ Laboratory of Nuclear Materials and Laboratory of Synchrotron Radiation and Femtochemistry Paul Scherrer Institut Villigen 5232 Switzerland

**Keywords:** additive manufacturing, machine learning, time‐resolved tomography, X‐ray imaging

## Abstract

Understanding three Dimensional (3D) fundamental processes is crucial for academic and industrial applications. Nowadays, X‐ray time‐resolved tomography, or tomoscopy, is a leading technique for in situ and operando 4D (3D+time) characterization. Despite its ability to achieve 1000 tomograms per second at large‐scale X‐ray facilities, its applicability is limited by the centrifugal forces exerted on samples and the challenges of developing suitable environments for such high‐speed studies. Here, Super Time‐Resolved Tomography (STRT) is introduced, an approach that has the potential to enhance the temporal resolution of tomoscopy by at least an order of magnitude while preserving spatial resolution. STRT exploits a 4D Deep Learning (DL) reconstruction algorithm to produce high‐fidelity 3D reconstructions at each time point, retrieved from a significantly reduced angular range of a few degrees compared to the 0–180° of traditional tomoscopy. Thus, STRT enhances the temporal resolution compared to tomoscopy by a factor equal to the ratio between 180° and the angular ranges used by STRT. In this work, the 4D capabilities of STRT were validated through simulations and experiments on droplet collision simulations and additive manufacturing processes. It is anticipated that STRT will significantly expand the capabilities of 4D X‐ray imaging, enabling previously unattainable studies in both academic and industrial contexts, such as materials formation and mechanical testing.

## Graphical abstract

A super time‐resolved tomography (STRT) approach is presented to reconstruct 4D X‐ray movies with an order‐of‐magnitude improvement in temporal resolution without sacrificing spatial resolution. By leveraging a physics‐informed deep learning algorithm that shares spatiotemporal features, STRT achieves high‐fidelity 3D reconstructions from sparse‐view, limited‐angle data, enabling the investigation of rapid dynamic processes beyond the reach of conventional tomoscopy.

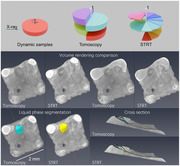

## 1 Introduction

X‐ray computed tomography (CT) is an essential tool across a plethora of fields in academia and industry for non‐destructive study of 3D structures.^[^ ^1^, ^2^, ^3^ ^]^ CT works by collecting a series of 2D images, or radiographs, as the sample rotates relative to the X‐ray source, covering an angular range between at least 0–180°.^[^ ^4^, ^5^ ^]^ These radiographs are then combined using computing algorithms to reconstruct a 3D volume.^[^ ^6^ ^]^ The high flux provided by large‐scale X‐ray facilities, like diffraction‐limited storage rings^[^ ^7^, ^8^ ^]^ and X‐ray Free‐Electron Lasers (XFELs),^[^ ^9^, ^10^ ^]^ opens the possibility to study not only matter in 3D but also to resolve their evolution in 4D (3D+time).^[^ ^11^, ^12^ ^]^ Specifically, time‐resolved X‐ray tomography, also known as tomoscopy, has revolutionized our understanding of dynamic systems through in situ, operando, and in vivo 3D characterization.^[^ ^13^, ^14^ ^]^ The demand for tomoscopy stems from the critical need for non‐destructive real‐time analysis of internal structures and processes in various disciplines, such as fractures in solids,^[^ ^15^, ^16^ ^]^ manufacture and assembly of products,^[^ ^17^, ^18^, ^19^, ^20^ ^]^ fast biological processes,^[^ ^21^, ^22^ ^]^ and product degradation in service.^[^ ^23^, ^24^ ^]^ For instance, understanding microstructural changes during manufacturing processes, such as alloy solidification, metal processing,^[^ ^25^ ^]^ and mechanical deformation,^[^ ^26^ ^]^ is critical for developing materials with enhanced properties. Traditional methods often fail to accurately capture these dynamic processes at the relevant spatiotemporal scale in a non‐destructive manner, highlighting the importance of tomoscopy as a crucial tool for studying such 3D processes.

Although tomoscopy is a well‐established 4D tool, it faces challenges in improving its temporal resolution.^[^ ^27^, ^28^ ^]^ Specifically, it is crucial when studying dynamic processes to optimize temporal resolution without significantly compromising other parameters such as spatial resolution, field of view, and the total possible acquisition period. The current approach to increase the temporal resolution in tomoscopy involves increasing the rotation speed. The fastest tomoscopy experiments use high‐speed rotation stages that enable rotations up to 500 Hz, which corresponds to one thousand tomograms per second.^[^ ^13^ ^]^ These acquisition speeds offer the opportunity to study processes such as alloy casting and sparkler burning.^[^ ^26^ ^]^ However, these high‐speed rotations induce centrifugal forces that are orders of magnitude greater than the gravitational acceleration, potentially altering the dynamics being studied. In addition, they complicate the fabrication of sample environments for in situ and operando characterization. Therefore, such fast rotations extremely limit the applicability of tomoscopy. An alternative approach for studying processes in 3D is X‐ray Multi‐Projection Imaging (XMPI), which can produce rotation‐free 3D movies^[^ ^29^, ^30^ ^]^ at framerates from kHz and beyond.^[^ ^31^, ^32^ ^]^ However, XMPI suffers from sparse angular acquisitions, which necessitate quasi‐reproducible experiments to retrieve accurate 3D movies. This hinders its applicability to complex and non‐reproducible dynamics.

Parallel to advances in 4D acquisition methods, significant improvements have been made in computational algorithms for image reconstruction. Historically, reconstruction algorithms can be categorized by the dimensionality of the solutions: from 2D slices to full 3D volumetric reconstructions, and, more recently, to dynamic 4D approaches that include the time dimension.^[^ ^33^, ^34^ ^]^ The most common reconstruction approaches for tomoscopy rely on reconstructing individual 2D slices for each time point using: i) analytical solutions such as filtered back projection,^[^ ^35^ ^]^ ii) iterative approaches,^[^ ^36^, ^37^, ^38^ ^]^ iii) compressed sensing,^[^ ^39^, ^40^, ^41^ ^]^ and iv) DL.^[^ ^42^, ^43^, ^44^ ^]^ DL approaches have significantly improved the reconstruction quality of 2D slices, offering solutions to challenges such as sparse‐view reconstruction and noise mitigation. Moreover, DL approaches have demonstrated the opportunity to provide direct 3D reconstructions for each time point, eliminating the need to stack 2D slices to retrieve the 3D volume. Examples of these approaches include Convolutional Neural Network (CNN) with regular grids or continuous function representations.^[^ ^45^, ^46^, ^47^ ^]^ However, CNN‐based methods can be computationally expensive, limiting the volume that can be reconstructed in 3D. More recent DL approaches based on implicit representations such as Neural Radiance Fields (NeRFs)^[^ ^48^, ^49^, ^50^ ^]^ have shown the ability to efficiently reconstruct large 3D volumes with X‐ray imaging. When it comes to 4D reconstruction, there is a limited selection of algorithms that can be applied to tomoscopy.

Among existing 4D reconstruction approaches, a prominent class is model‐based iterative reconstruction (MBIR) techniques,^[^ ^51^ ^]^ as well as their more recent extensions for dynamic imaging.^[^ ^28^, ^52^, ^53^, ^54^ ^]^ These methods typically incorporate spatial and temporal priors, often through sparsity‐promoting regularization or total variation constraints, to reconstruct time‐resolved volumes from undersampled projection data. While such methods are capable of handling sparse‐view and limited‐angle scenarios, they generally require the sample to undergo a complete 180‐degree rotation to reconstruct a single tomographic volume. This requirement fundamentally constrains the achievable temporal resolution. Moreover, these approaches often rely on voxel‐based grid representations and iterative solvers, which result in substantial computational and memory overhead. Their temporal modeling is typically discrete, either frame‐based or event‐driven, and lacks a continuous, unified representation over time. These limitations make it challenging to apply such methods to fast‐evolving or non‐reproducible dynamic processes, particularly in scenarios demanding high temporal resolution or real‐time performance. In recent years, 4D‐DL computer vision approaches have shown the possibility of capturing complex 4D scenes in a computationally efficient manner, such as K‐planes,^[^ ^55^ ^]^ D‐NeRF,^[^ ^56^ ^]^ and Hexplane.^[^ ^57^ ^]^ Despite their potential, only a few of these algorithms have recently been adopted by the X‐ray community, such as 4D‐ONIX,^[^ ^33^ ^]^ designed explicitly for XMPI. These 4D X‐ray approaches^[^ ^33^, ^58^ ^]^ offer an opportunity to solve tomoscopy directly in its intrinsic 4D space, thereby i) simplifying the reconstruction process, ii) imposing spatiotemporal consistency, and iii) offering opportunities to include 4D physical constraints. Thus, the combination of such algorithms together with state‐of‐the‐art tomoscopy acquisition systems offers an opportunity to push the spatiotemporal resolution of 4D X‐ray imaging.

In this paper, we present STRT, a novel approach that enhances the temporal resolution of tomoscopy while preserving the spatial resolution. The framework is self‐supervised and case‐specific, requiring no pre‐training, external datasets, or prior knowledge beyond the measured radiographs, which makes it broadly applicable and experimentally practical. This is achieved by reducing the angular range required to retrieve a 3D reconstruction for each temporal instance of the studied dynamics. STRT exploits a deep learning approach to produce high‐quality 4D reconstructions of dynamic objects using very few projections or radiographs within a narrower angular range than the current angular range required by tomoscopy: 0–180°. This results in a temporal resolution enhancement equal to the ratio between the angular range used by STRT and 0–180°. We demonstrate, using both simulated and experimental data, that STRT can improve the temporal resolution by at least one order of magnitude compared to conventional tomoscopic experiments, while preserving the spatial resolution. Such improvement stems from the possibility of reconstructing tomoscopic data in its intrinsic dimension (4D) and sharing features across space and time. Consequently, we envision STRT as an enabling tool that expands the possibilities of tomoscopy, not only by accessing academic and industrial processes previously inaccessible due to centrifugal forces or limited sample environments at large‐scale X‐ray facilities but also by enhancing the temporal resolution of laboratory equipment to bring tomoscopy into standard lab applications.

## 2 Super Time‐Resolved Tomography

**Figure 1:**
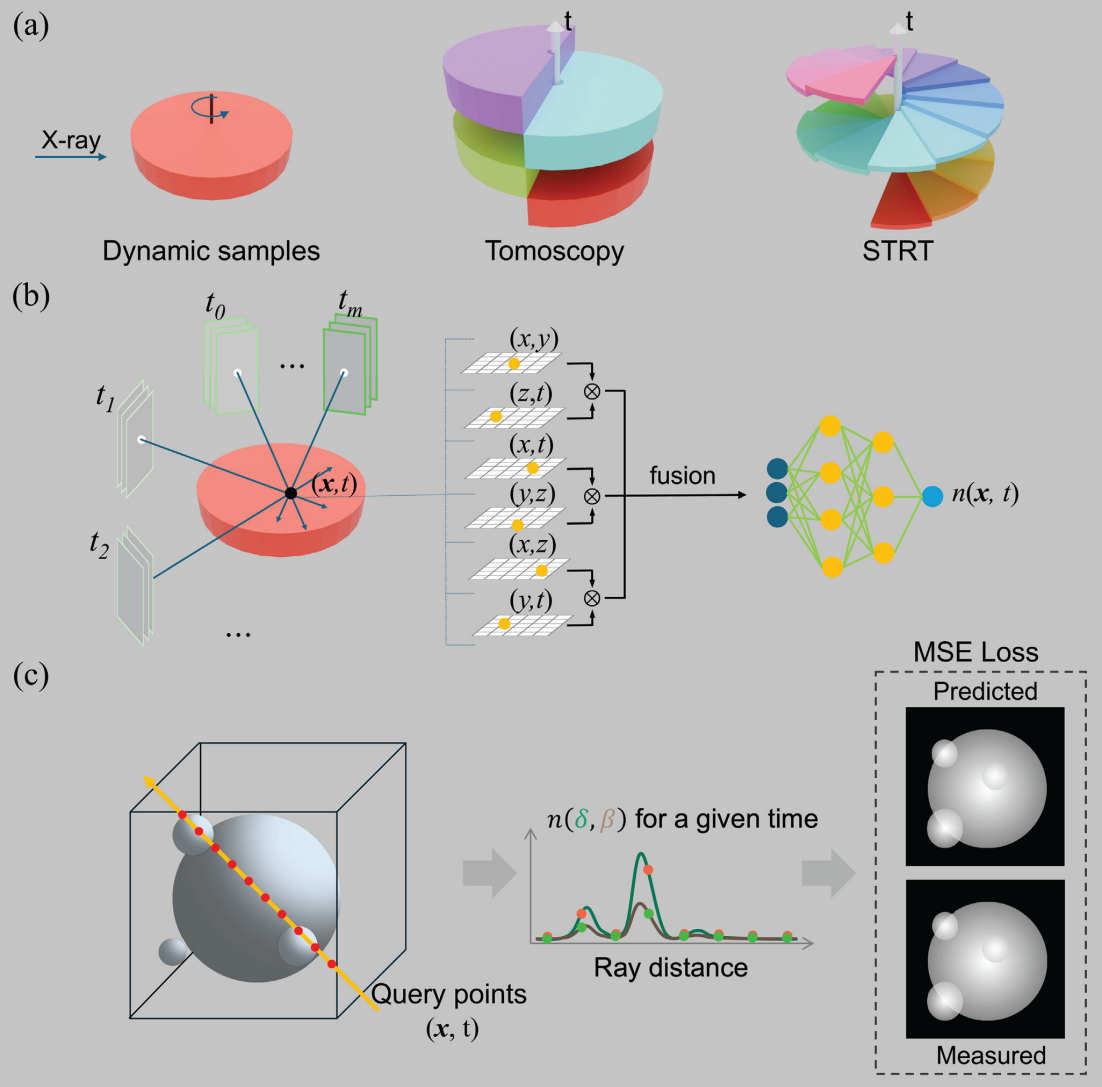
STRT concept. a) Data acquisition of STRT vs. tomoscopy. The thickness of each sector represents the time period during which the samples are assumed to be static. Tomoscopy requires a full 180‐degree scan to capture a single time point, while STRT reduces the required scan angle to much less than 180° by exploiting spatiotemporal redundancies and sharing information across space and time. b) X‐Hexplane tensorial model. X‐Hexplane contains six feature planes spanning each pair of coordinate axes (e.g., XY, ZT). Points in spacetime are projected to each plane. The features extracted from the six planes are fused and sent to a tiny Multilayer Perceptron (MLP) to get *n*(**x**, *t*) at a specific spatiotemporal point. c) X‐Hexplane rendering and cost function. 2D projections from a given experiment angle and time point can be rendered by integrating *n*(*x*, *t*) along the ray direction at that specific time point. The parameters of X‐Hexplane are updated by minimizing the Mean Squared Error (MSE) loss between the predicted results and the measured projections.

State‐of‐the‐art tomoscopy provides 4D information by reconstructing each time instance independently of a dynamical process from a complete series of radiographs acquired over an angular range of 0–180°, see **Figure** **1**a. This acquisition approach, including an incremental reconstruction method,^[^ ^59^ ^]^ which shifts the start of each 180° range by one projection, derives from conventional tomoscopy methods that reconstruct 2D slices and stack them into a 3D volume. In contrast, intrinsic 4D reconstruction approaches offer opportunities to increase temporal resolution by relaxing the acquisition protocols, exploiting 4D consistencies in the data, and simplifying both the reconstruction and analysis processes.

Our approach, christened STRT, enhances the temporal resolution of tomoscopy by utilizing a smaller angular range per time point compared to tomoscopy. For this angular range, the sample is assumed static, and the corresponding projections are labeled with the same timestamp for reconstruction purposes. The acquisition concept for STRT is depicted in Figure 1a. STRT uses a continuous rotation for the acquisition as done with tomoscopy, but each time point is defined over a smaller angular range, i.e., less than 180° rotation. This provides temporal enhancement that is proportional to the ratio between the angular range used in STRT and 0–180° used in tomoscopy experiments. To enhance temporal resolution while preserving spatial resolution, STRT utilizes a 4D reconstruction framework that shares information between space and time throughout acquisition, thereby alleviating the angular range requirement. In contrast, state‐of‐the‐art CT reconstruction methods provide only 2D reconstructed slices for a specific time point.

Our 4D DL reconstruction framework, X‐Hexplane, builds upon the Hexplane^[^ ^57^ ^]^ architecture, which utilizes a tensorial data structure to represent dynamic 4D volumes,^[^ ^60^ ^]^ and takes angle‐ and time‐resolved radiographs as input. Unlike Hexplane, designed for visible light, X‐Hexplane incorporates essential adaptations for X‐ray tomography and imaging, including a physics‐based forward model and the prediction of the complex index of refraction field *n*(**x**, *t*), rather than RGB appearance. The output of the network is physically meaningful, as it encodes both phase and absorption components, which are critical for simulating realistic X‐ray measurements. To model X‐ray projection from dynamic volumes, we extend the multiplane representation with a physics‐based forward model based on the projection approximation,^[^ ^61^ ^]^ which assumes that the X‐ray wavefront is only weakly perturbed as it traverses the sample (weak‐scattering approximation). This provides a physical‐forward model to generate projection images (radiographs) by integrating along rays through the predicted volume. This physical‐forward model can be extended beyond the weak‐scattering approximation by using, e.g., multi‐slice and propagation‐based methods.^[^ ^61^ ^]^ X‐Hexplane describes a 3D dynamic sample in terms of the index of refraction at each spatial‐temporal point *n*(**x**, *t*). The index of refraction quantifies the X‐ray interaction with matter and is represented as *n* = 1 − δ + *i*β,^[^ ^61^ ^]^ where i) the real part δ captures the elastic interactions that result in wave phase shifts, offering high sensitivity to structural variations even in low‐Z materials via the so‐called phase contrast,^[^ ^62^ ^]^ and ii) the imaginary part β relates to the inelastic interactions such as X‐ray photon absorption and is related to the attenuation contrast.^[^ ^1^, ^63^ ^]^

To provide a 4D computationally efficient reconstruction, X‐Hexplane represents a dynamic process as an explicit voxel grid of features and reduces memory consumption by tensorial factorization,^[^ ^57^, ^60^ ^]^ as depicted in Figure 1b. Specifically, X‐Hexplane decomposes a 4D spacetime grid into six feature planes spanning each pair of coordinate axes, (XY, ZT), (XZ, YT), (XT, YZ). First, X‐rays are marched from each pixel of the captured images, and points are randomly sampled along the rays. Each (**x**, *t*) sampled point on the left‐hand side of Figure 1b is projected onto the six feature planes. Bilinear interpolation is applied to extract six corresponding feature vectors, which are then aggregated to form a fused feature vector. Subsequently, a lightweight MLP, a simple feedforward neural network composed of fully connected layers, is employed to regress the fused vector and predict the value of the index of refraction at any given spatiotemporal point, *n*(**x**, *t*). See the Experimental Section for a more detailed description of the X‐Hexplane algorithm. Once a differentiable description of the *n*(**x**, *t*), with two output channels representing the real (δ) and imaginary (β) parts of the index of refraction, as a function of the input projections is provided, we can generate projections along any X‐ray propagation direction and time by integrating the point values along the X‐ray propagation direction using the projection approximation. In this work, a fixed distance from the detector and a parallel beam geometry are used to compute the projections. Nonetheless, by modifying the geometric expression, cone‐beam or fan‐beam can also be implemented to account for different experimental configurations. Finally, to optimize X‐Hexplane, we minimize an MSE cost function between the predicted projections with X‐Hexplane at a given time and the corresponding measured projections at the same time point. This minimization process depicted in Figure 1c can be seen as an extension to 4D of current minimization approaches used in iterative reconstructions, such as algebraic reconstruction techniques for 2D slice reconstructions.^[^ ^36^ ^]^

To sum up, X‐Hexplane provides a 4D X‐ray reconstruction framework that enables STRT by i) incorporating the physics of X‐ray propagation into the model, ii) using a tensorial representation of the dynamics process to reduce memory footprint, and iii) sharing the features over space and time to solve the sparse view, limited angle problems.

### 2.1 STRT Performance Evaluation

To evaluate and demonstrate the potential of the proposed X‐Hexplane, we required accurate ground truth data. Therefore, we utilized simulated 4D data and standard tomoscopy experiments instead of STRT experiments. The ground truth for the former was derived from the 4D simulated data, while for the latter, it was obtained from standard tomoscopy reconstructions at each time point using TomoPy.^[^ ^64^ ^]^

To simulate an STRT experiment from tomoscopy data, we developed a specialized data extraction that uses different continuous angular ranges for each time point. Specifically, for each time point in STRT, we selected projections that span a fixed angular range within a 360° rotation to ensure continuous data acquisition over time. For instance, at time point *t*_0_, projections covered an angular range from 0° to θ°, extracted from the first 360° rotation of the tomoscopy data. At time point *t*_1_, projections covered θ° to 2θ°, extracted from the second 360° rotation of the tomoscopy data, and so on. It should be noted that we extracted each time point from a 360° rotation instead of the typical 180° rotation used in tomoscopy. This approach was chosen to avoid 4D inconsistencies that arise when combining 180° data due to the flipping requirement for two consecutive time points, which can hinder the quality of X‐Hexplane reconstructions. We emphasize that this data selection does not reflect a requirement of the STRT acquisition scheme itself but rather a constraint imposed by using tomoscopy data as a ground‐truth reference. It should be noted that an STRT experiment would use all the continuous frames acquired through a continuous rotation. Then, the reconstruction method would be applied directly to the recorded projections, without requiring any data selection or a full or half‐rotation dataset.

**Table** **1** reports the different angular ranges and projections data used in our validation experiments for tomoscopy and STRT, together with the temporal enhancement (TE) between STRT and tomoscopy. To assess the resolution for a given temporal enhancement, we compared the quality of the STRT reconstructions with the ground truth as a function of the angular range employing the Fourier Shell Correlation (FSC).^[^ ^65^ ^]^ The FSC calculates the normalized cross correlation between the reconstructions and the ground truth in frequency space over shells. Therefore, it provides a robust metric for comparing the spatial frequency content of the two results, ensuring a thorough evaluation of the neural network's performance for each time point. Here, we used FSC with the half‐bit threshold criterion to determine the achievable resolution.^[^ ^65^ ^]^ In addition to FSC, we also computed three widely used image quality metrics, Peak Signal‐to‐Noise Ratio (PSNR), Structural Similarity Index Measure (SSIM), and Normalized Root Mean Square Error (NRMSE), to provide a more comprehensive and quantitative evaluation of reconstruction fidelity. These complementary metrics enable cross‐validation of structural consistency and intensity accuracy across varying temporal enhancement conditions. The results for each process are summarized in Table 1.

**Table 1:** Result evaluation for the simulated and experimental datasets as a function of the temporal enhancement (TE). The columns list the number of timesteps (No. ts) and the angular range per timestep (Δθ) for both tomoscopy and STRT, along with the temporal enhancement factor of STRT relative to tomoscopy. The final columns report the mean and standard deviation of the evaluation metrics computed over the entire time sequence.

	**Tomoscopy**		**STRT**			**Reconstruction Metrics**			
Process	No. ts	Δθ	No. ts	Δθ	TE	FSC	PSNR	SSIM	NRMSE
Droplet collision	75	180°	75	3.0°	60×	4.1 ± 1.8	40.67 ± 2.67	0.977 ± 0.012	0.009 ± 0.002
				9.0°	20×	3.0 ± 0.4	45.16 ± 2.84	0.990 ± 0.002	0.006 ± 0.002
				18.0°	10×	2.8 ± 0.3	49.00 ± 2.60	0.990 ± 0.002	0.004 ± 0.001
Additive manufacturing	200	180°	200	0.9°	200×	5.7 ± 0.8	14.26 ± 1.14	0.696 ± 0.008	0.195 ± 0.026
				9.0°	20×	3.7 ± 0.6	22.10 ± 1.22	0.738 ± 0.013	0.079 ± 0.012
				18.0°	10×	3.2 ± 0.5	22.80 ± 0.80	0.765 ± 0.012	0.073 ± 0.007

In the following sections, we provide a detailed description of our validation results for both simulated and experimental data. The specific network and training details are provided in the Experimental Section.

### 2.2 Simulation Results

We first assessed the performance of STRT using 4D simulated datasets describing a coalescence process as a result of a binary droplet collision.^[^ ^66^, ^67^, ^68^ ^]^ This 4D process was modeled using Navier‐Stokes Cahn‐Hilliard equations.^[^ ^33^, ^69^ ^]^ To be consistent with a potential STRT experiment on such a system, we assumed that the X‐ray source was continuously rotated instead of the sample.^[^ ^70^, ^71^ ^]^ For each of the 75 simulated time points in this process, projections with 128 × 128 pixels were generated. The modeled data was used to simulate an STRT experiment. We extracted continuous subsets of projections that cover angular ranges of 3°, 9°, and 18° per time point for training, which corresponded to 60×, 20×, and 10× temporal enhancement, respectively.

**Figure 2:**
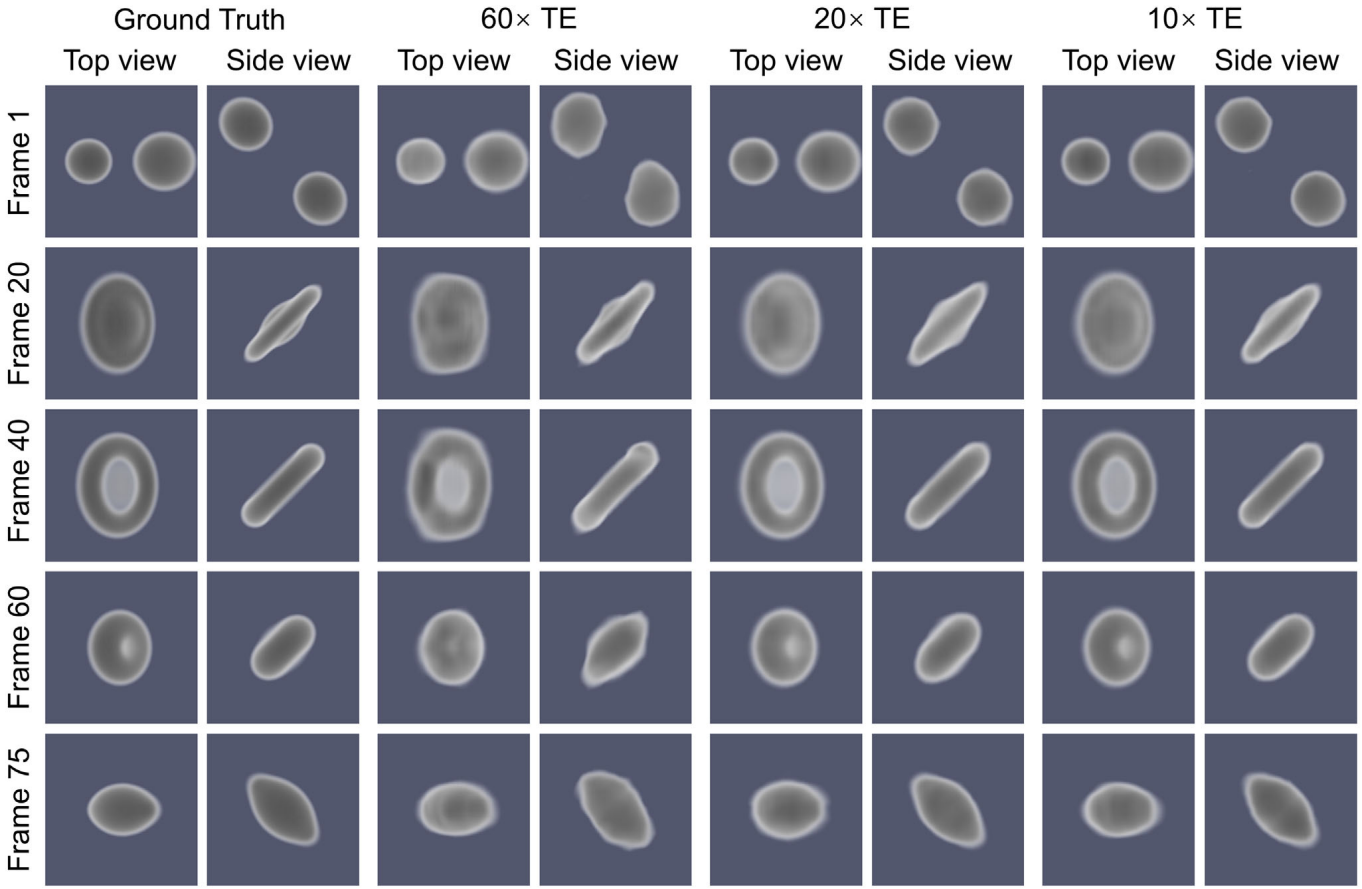
STRT reconstructions for droplet collisions compared to the ground truth. The left column depicts the 4D ground truth for different time points (frames) from two orthogonal views: one along the projection axis (side view) and one perpendicular to this plane (top view). The other columns show the corresponding STRT reconstructions for 60×, 20×, and 10× temporal enhancement (TE).

**Figure 3:**
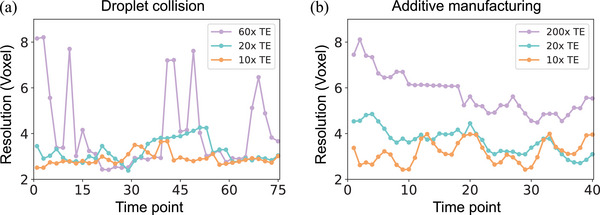
Spatial resolution determined by FSC as a function of time. a) illustrates the resolution changes during droplet collision with temporal enhancements (TE) of 60×, 20×, and 10×. b) presents the evolving revolution in additive manufacturing over time with temporal enhancements of 200×, 20×, and 10×.

**Figure** **2** presents the ground truth and reconstruction volumes as a function of the temporal enhancement from two orthogonal views at specific time points. A side view along the projection plane and a top view perpendicular to it were selected. The top view is particularly challenging to reconstruct, as it aligns with the rotation axis and is perpendicular to the incident X‐rays. Additionally, Video S1 (Supporting Information) provides a video that shows a comparison between ground truth and STRT reconstruction results as a function of the temporal enhancement. The quantitative reconstruction evaluation via the FSC is summarized in Table 1. As the angular sampling range increases from 3.0° to 18.0°, the FSC mean values gradually improve, indicating enhanced structural fidelity under lower temporal enhancement factors. This trend is also reflected in the complementary metrics: PSNR increases from 40.67 to 49.00 dB, SSIM improves from 0.977 to 0.990, and NRMSE decreases from 0.009 to 0.004. These results confirm that even under extreme angular undersampling conditions (e.g., 60× TE), the proposed method is able to recover the main features of the dynamic process, while progressively improving reconstruction accuracy as more angular information becomes available. The FSC as a function of time for the binary droplet collisions is shown in **Figure** **3**a. For the reconstructions with an angular range of just 3°, i.e., a temporal enhancement factor of 60, the reconstructed 3D movie achieved an average resolution of 4.1 voxels, only two times worse than the Nyquist‐limited resolution of two voxels. In the 20× and 10× cases, the results successfully capture both shapes and dynamics across all frames, achieving an average resolution of less than three voxels almost compatible for all the time points with the Nyquist resolution.

### 2.3 Additive Manufacturing Results

X‐ray tomoscopy experiments on laser‐based powder bed fusion (LPBF) of iron oxide doped alumina were performed at the TOMCAT beamline of the Swiss Light Source. The tomoscopy presented in this work was performed while scanning the sample with a speed of 1 mms^−1^ using a green (532 nm) pulsed laser (1.5 ns pulse duration) with power set to 20 W. The observed process was remelting of the previously deposited layer, meaning that in this case, no new powder layer was deposited in the sample and the laser interacted directly with the solidified ceramics. The detailed description of the used material and the setup for the operando tomography during the LPBF process can be found in Ref. [72]. The studied LPBF process consists of two stages. The first involves a powder layer deposition. In the second stage, laser scanning a selected region of the sample induces localized melting of the powder, which is followed by the cooling down and solidification of the molten material. By adding consecutive multiple layers, a stable, 3D structure is obtained. The morphology and microstructure of LPBF‐manufactured materials are strongly linked to the dynamics of the melt pool. To track the behavior of the molten volume and the solidification processes, tomoscopy measurements with an acquisition rate of 100 tomograms per second were recorded, each with 200 projections over 180°. This high temporal resolution was achieved using the high polychromatic flux provided by the TOMCAT superbending magnet source. Due to the limited temporal (broad spectrum) and spatial (large source size) coherence of the TOMCAT source, limited phase contrast was observed in the projections. Thus, we performed phase‐retrieval using transport of intensity equations for homogeneous objects,^[^ ^73^ ^]^ which resulted in a single reconstructed component δ, while the β (attenuation) is not retrieved. Our X‐Hexplane architecture was, therefore, tailored for this experiment to be sensitive only to the phase‐retrieved component of the index of refraction. The projections or radiographs were acquired with the GigaFRoST camera system.^[^ ^74^ ^]^ The camera system provided an effective pixel size of 2.75 µm in this experiment, with radiographs measuring 160 × 1056 pixels for additive manufacturing. To enhance visualization and accelerate the reconstructions, the top part of the object was selected, and the width was scaled down, resulting in a final projection size of 70 × 528 pixels. Moreover, we selected the 200 time points where the printing process took place. Datasets were prepared with different temporal enhancements of 200×, 20×, and 10×, corresponding to angular ranges of 0.9°, 9°, and 18° per time point, respectively. The specific details of the dataset processing are discussed in the Experimental Section.

**Figure 4:**
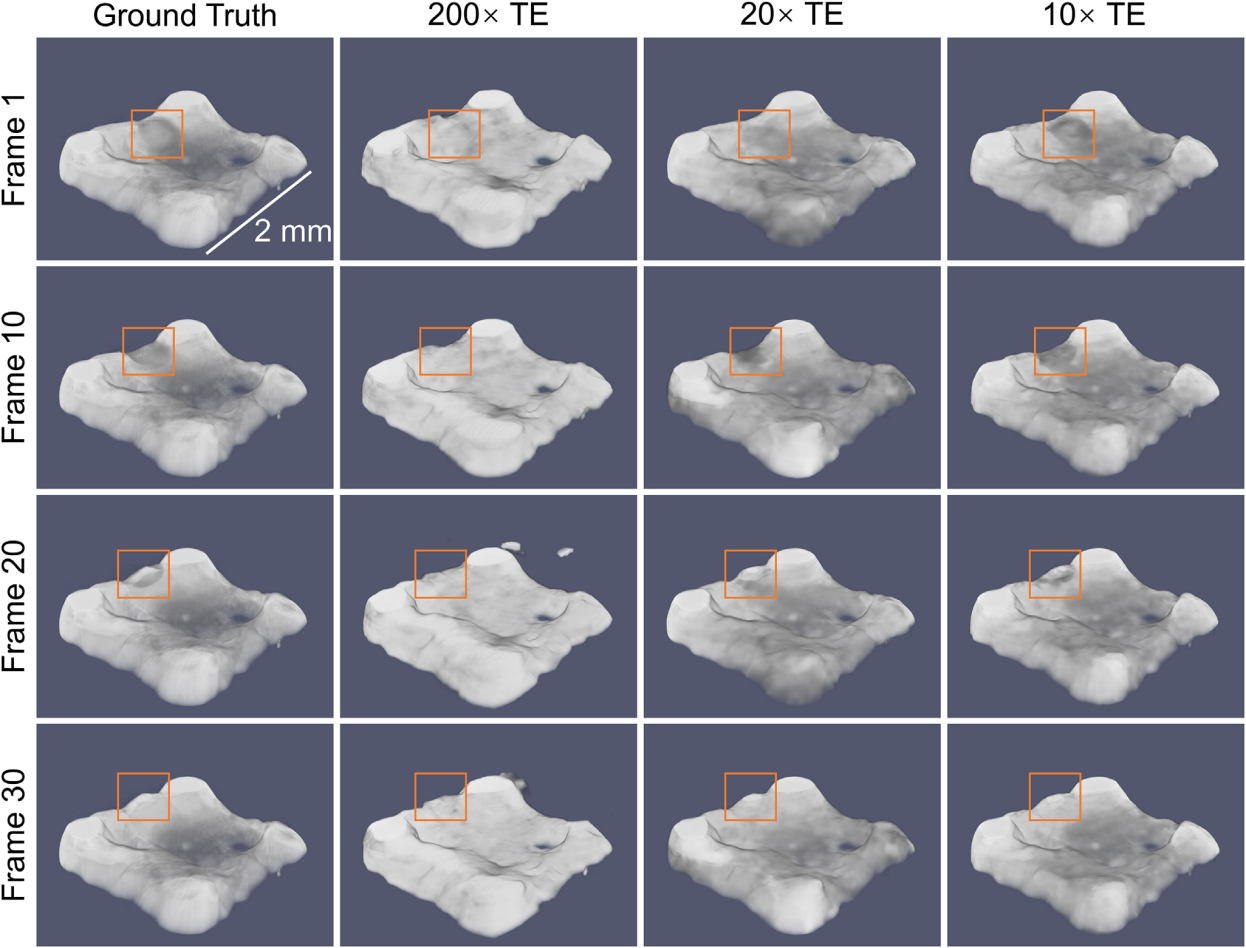
STRT reconstructions for additive manufacturing and ground truth. The ground truth and STRT results for 200×, 20×, 10× temporal enhancement (TE) are displayed in separate columns, while different rows represent distinct time frames of the printing process. The dynamics of the printing processes are marked by solid squares.

**Figure** **4** presents the comparison between the ground truth obtained from full 180° per time point and the corresponding STRT reconstructions. The different rows in the figure depict the sample's state at various time points. A video comparing the reconstructions and the ground truth is provided in Video S2 (Supporting Information). As it can be observed in Figure 4, STRT produces promising results even with only 1 projection per time point, effectively capturing the general shape of the sample. However, noticeable artifacts and distortions appear, particularly in finer structures in the area that is being printed (orange squares in Figure 4). The reconstruction corresponding to 20× temporal enhancement improved overall shape preservation but still struggles with accurately recovering the finer details of the printing process. With 10× temporal enhancement, both deformation stages were successfully reconstructed, indicating that an angular range of 18° per time point is sufficient to capture the dynamic process. The mean and standard deviation of the metrics evaluation are reported in Table 1. FSC values decrease from 5.7 ± 0.8 under 200× temporal enhancement to 3.2 ± 0.5 under 10× TE, indicating progressively improved structural fidelity with increasing angular coverage. This trend is corroborated by the PSNR, SSIM, and NRMSE metrics, which show substantial quality gains as the angular range increases: PSNR rises from 14.26 to 22.80 dB, SSIM improves from 0.696 to 0.765, and NRMSE drops from 0.195 to 0.073. These results demonstrate the ability of STRT to recover meaningful structure even under severe undersampling, while clearly benefiting from additional angular information. The FSC as a function in time is shown in Figure 3b. Such results confirm that the best results are achieved with 10× enhancement with a resolution of approximately three voxels compared to the ground truth.

**Figure 5:**
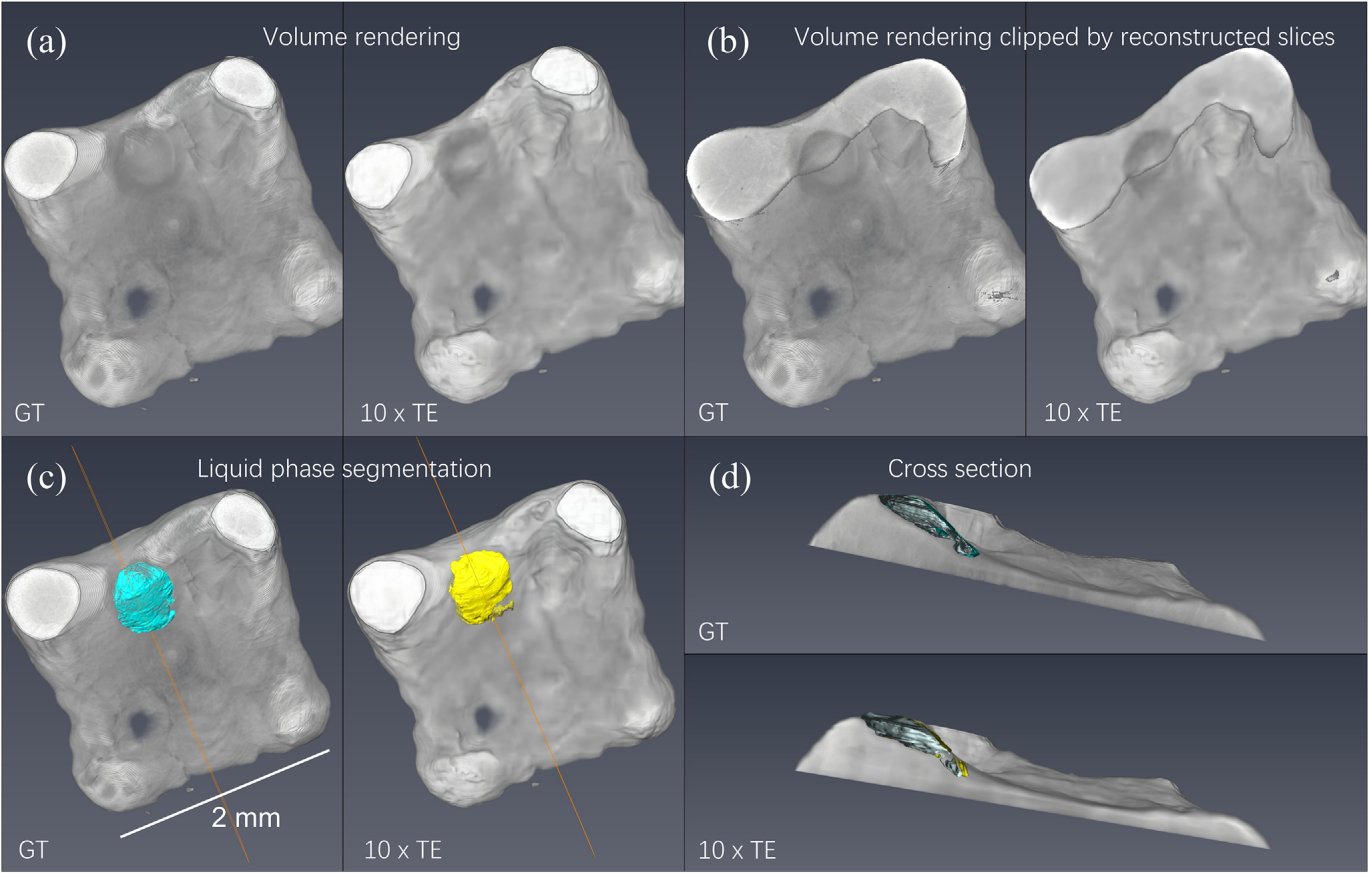
Comparison of the reconstruction performed with 10× temporal enhancement (TE) with the ground truth a) volume rendering, b) volume rendering clipped by slices reconstructed using corresponding approaches (ground truth and 10× TE), c) results of material segmentation highlighting liquid surfaces, d) cross‐sections along the orange line indicated in (c).

For further evaluation of the quality of the obtained reconstructions and to assess their applicability for the studies of the LPBF process, material segmentation was performed using standard tools available in the commercial software Thermo Scientific Avizo. The results are illustrated in **Figure** **5**. Figure 5a,b shows the comparison of the reconstructed volume obtained with 10× enhancement with the ground truth by volume rendering and the obtained tomographic slices, clipping the same volumes approximately through the middle of the melt pool depth. A simple image contrast adjustment allows an obvious distinction between the solid and liquid phases, which in turn facilitates the material segmentation. The liquid surface generated based on the segmentation is displayed in Figure 5c (top view) and d) (cross‐section) and clearly shows the similar shape of the obtained melt pool in both cases. The volume of the molten phase obtained for the ground truth reconstruction was 1.4× 10^6^ µ*m* ^3^, and for 10× temporal enhancement it was 1.6× 10^6^ µ*m* ^3^. This difference is within the possible deviation range due to the obtained spatial resolution.

## 3 Discussion and Conclusion

STRT has demonstrated the potential to significantly enhance the temporal resolution of tomoscopy in both simulated and experimental cases, achieving at least a tenfold improvement while preserving spatial resolution. The highest achievable temporal enhancement with STRT while preserving the tomoscopy spatial resolution depends on the specific dynamics being studied. Thus, we discuss each test independently.

In the case of droplet collision, as shown in Figure 3a, intermediate collision states are more challenging to reconstruct across all temporal enhancement levels due to the increased complexity of the dynamics. The degradation in reconstruction quality is particularly evident at 60× enhancement, where the resolution is lower, and the reconstructed structures fail to capture the precise morphology of the droplets. This suboptimal performance can be attributed to the limited angular coverage for each time point, which is more dramatic for abrupt changes in transient states. Specifically, for the 60× temporal enhancement, the total angular coverage through the STRT is restricted to 75 × 3° = 225° while having the same number of time intervals as the 20× and 10× temporal enhancement cases. This limited information provided to the network makes the transient state even more challenging to reconstruct. In contrast, at 10× and 20× enhancement, the reconstructions successfully capture both fine and coarse structural details across all frames. In Figure 3a, the 10× temporal‐enhancement curve appears more stable with fewer fluctuations, whereas the 20× enhancement curve shows greater variability at transient stages. This demonstrates that the larger angular range per time point provides high‐quality spatiotemporal reconstructions even in the presence of fast dynamics with respect to the acquisition framerate.

The best resolution reconstruction for the laser powder bed fusion tomoscopy is achieved at 10× enhancement but deteriorates significantly at 200× enhancement as depicted in Figure 3b. The 200× enhancement fails to accurately reconstruct the morphology of the laser‐processed part and to provide a clear contrast between the liquid and solid phase, with an average resolution of 6.9 voxels over the first ten time points. This degradation mainly arises from the extremely limited angular coverage per time point—only 0.9° per time frame, leading to a total angular coverage of 200 × 0.9° = 180° over the full 4D acquisition. The restricted angular information at individual time points and across the whole acquisition sequence hinders the algorithm's ability to retrieve 4D shareable information effectively, particularly in regions with complex geometries and rapid dynamics. As a result, it is not possible to image structural defects such as cracks and pores appearing in the sample nor to analyze the dynamics of the molten phase since it cannot be distinguished from the solid phase. For the 20× case, although the reconstruction still struggles to precisely capture the phenomena occurring in the region interacting with the laser and therefore changing over time (with an average resolution of 4.2 voxels in the first ten time frames), it successfully captures the overall shape of the manufactured alumina part across all frames, representing a significant improvement in FSC results. Finally, the 10× enhancement results provide a better and more stable FSC resolution over time. The improved reconstructions at 10× and 20× enhancement compared to the 200× are attributed to the larger angular range per time point, which provides more angular information with limited time points. This additional information enhances the algorithm's capability to reconstruct both fine and coarse structural features with greater accuracy. Such results demonstrate that STRT successfully retrieves the dynamic process consisting of the 3D reconstructions for each time point depicted in Figure 4, providing a sufficient contrast between the observed phases (solid and liquid). This is clearly demonstrated in Figure 5, presenting the results of material segmentation. Both the shape and the volume of the melt pool evaluated from the reconstruction based on 10× temporal enhancement were in good agreement with the ground truth. It should be noted that in the case of the studied material, namely, alumina, the contrast between solid and liquid is inherently low. Therefore, this data set, though very relevant for real applications, is a particularly challenging case.

Although STRT offers clear advancements over tomoscopy in additive manufacturing, the data selection and processing required to compare STRT with tomoscopy as a ground truth introduces artifacts that limit the full potential of STRT. For example, one should notice that the resolution curve in Figure 3b for 10× enhancement exhibits a periodic pattern with a 20‐time‐step interval, which arises from the fact that projections are acquired from the same viewing angle of the sample every 360° / 18° = 20 time steps. These periodic peaks in the curve result from noise and inconsistencies between the alignment of projections when interpreting tomoscopy data as STRT. Another key effect that causes such inconsistencies is edge enhancement due to X‐ray propagation. Edge enhancement effects refer to the phenomenon where edges of structures appear more pronounced due to phase shifts in the transmitted wavefront, leading to interference effects that enhance contrast at sample boundary regions. When viewed from certain angles, one boundary of the object may overlap with another boundary region, enhancing this effect. While phase retrieval methods^[^ ^73^ ^]^ can remove the edge enhancement effect from certain projections, they can introduce errors in projections that contain overlap regions, leading to inconsistencies between different projections together with resolution loss. These inconsistencies can affect the accuracy of the STRT reconstructions, particularly in regions with complex geometries or fine structural details. As such overlap is produced in a periodic manner due to the rotation, one can observe this periodicity in the peaks of the FSC curve.

In spite of the STRT improvements in temporal resolution compared to state‐of‐the‐art methods, several challenges and limitations remain. The algorithm relies on resolving features at each spacetime point, making it less effective for datasets with high noise levels or faint structures. In such cases, the achievable temporal resolution is constrained by the photon flux per projection, as insufficient contrast can degrade the model's ability to reconstruct dynamic features. This limitation is governed by the Rose criterion,^[^ ^75^ ^]^ which defines the minimum contrast‐to‐noise ratio required to detect a feature. For both phase contrast and absorption imaging, the resolvability of relevant features can be quantitatively assessed using the signal‐to‐noise ratio and contrast‐to‐noise ratio criteria, as discussed in previous studies.^[^ ^76^, ^77^ ^]^ Ensuring adequate photon flux per projection is therefore essential for high‐fidelity reconstruction, especially when pushing to higher temporal enhancement factors. Moreover, in high temporal enhancement STRT experiments, dynamical processes with abrupt changes occurring within only a few projections or frames present significant challenges, as the limited angular information available for sharing across the time sequence is insufficient to accurately capture these rapid transitions. Increasing the number of time points (framerate) while ensuring minimal sample changes between consecutive frames can further improve reconstruction quality at higher temporal enhancements. This approach requires a fast‐framerate camera and a high‐flux X‐ray source to leverage the information‐sharing capabilities of STRT and compensate for the missing angular views. Additionally, transferring knowledge across different experiments on the same or similar dynamical processes can serve as an effective strategy to mitigate such an effect.^[^ ^33^ ^]^ By conducting multiple experiments and optimizing them collectively, the model can learn shared features across not only 4D but also different experiments, leading to better convergence and improved accuracy in the reconstruction results. Finally, incorporating the physics of the studied dynamics to further constrain the process can also enhance spatiotemporal knowledge sharing,^[^ ^78^, ^79^ ^]^ thereby mitigating limitations and improving reconstruction fidelity.

To conclude, we have introduced STRT, a 4D X‐ray imaging approach that enhances the temporal resolution of the state‐of‐the‐art 4D X‐ray imaging technique (tomoscopy) by exploiting a new acquisition approach and a self‐supervised 4D DL framework. Compared to tomoscopy, STRT enhances the temporal resolution by at least an order of magnitude while preserving spatial resolution, addressing the limitations imposed by centrifugal forces and the challenges of developing suitable environments for 4D high‐speed studies with X‐ray imaging. By exploiting a 4D DL reconstruction algorithm, STRT achieves this enhancement through several key advancements: i) incorporating the physics of X‐ray propagation into the model, ii) using a tensorial representation of the dynamics process to reduce memory footprint, and iii) sharing the features over time to solve the sparse view problems. We have demonstrated the capabilities of STRT through proof‐of‐concept experiments on both simulated and experimental data, where high‐quality reconstructions were achieved with a temporal resolution enhancement of at least a factor of ten without compromising spatial resolution. The ability to capture these rapid phenomena with a tenfold increase in temporal resolution enables a more detailed understanding of transient dynamics, defect formation, and structural evolution in such systems. Specifically, in the case of the demonstrated example of the laser powder bed fusion studies, the increase in temporal resolution will allow the scientific community to broaden the range of studied materials. In this process, the scanning speed of the laser determines the speed of the melt pool translation. In the case of ceramics, the scanning laser speed is typically low (several to several tens of mm per s), and therefore, tomoscopy with a temporal resolution of 100 tps allowed tracking the melt pool dynamics in alumina.^[^ ^72^ ^]^ However, for metals, the laser speed is typically one or two orders of magnitude higher, and therefore, conventional tomoscopy would not be possible to apply due to the need to increase the rotation speed. Thus, the possible gain in temporal resolution presented in this work can enable 4D imaging of LPBF of materials, which was not possible with the previously used methodology. This type of experimental data will be crucial for understanding the physical phenomena determining the microstructure and quality of the additively manufactured materials, but also will provide information necessary for validation and development of numerical models for additive manufacturing processes, which was highlighted in Ref. [80]. Moreover, we envision the combination of STRT with other time‐resolved 3D techniques such as XMPI to further boost the temporal resolution and 4D accuracy of such approaches. To sum up, the significant enhancement in temporal resolution achieved with STRT opens new possibilities to investigate industrially and scientifically relevant processes that were previously inaccessible with tomoscopy and other advanced 4D X‐ray imaging methods. STRT will not only be impactful at large‐scale X‐ray facilities by enabling 4D imaging at rates exceeding 1000 tomograms per second, but also enhance 4D imaging using X‐ray laboratory sources.

## 4 Experimental Section

### X‐Hexplane Algorithm

The implementation of X‐Hexplane, a 4D reconstruction framework that models the complex spatiotemporal refractive index field *n*(**x**, *t*) as a continuous function over space and time, is described. This function was parameterized by a coordinate‐based neural network, following the X‐Hexplane architecture, which factorizes the 4D domain into a set of low‐rank 2D feature planes to enhance memory efficiency and learning capability. Each plane stores a learnable embedding defined over a specific coordinate pair, capturing local spatial and temporal correlations. Specifically, the spatial planes (XY, XZ, YZ) encode the static structural features that were shared across all time steps, enabling the model to retain consistent geometric information. In contrast, the spatiotemporal planes (XT, YT, ZT) capture the temporal evolution of features at each spatial location, allowing the network to modulate the static structure in time and accurately represent dynamic changes. At any given spatiotemporal coordinate, the model queries the corresponding positions on all six feature planes, retrieves the associated feature vectors via interpolation, and concatenates them into a unified latent representation. This combined representation was then decoded by a MLP to regress the refractive index at that spatiotemporal point. By jointly encoding spatial structures and temporal evolution, this design allows the network to share information across different time steps, enabling robust reconstruction of dynamic scenes even under severe angular undersampling. The embedded features were optimized end‐to‐end through supervision with a physics‐based X‐ray forward model, ensuring that the learned representations remain physically consistent with observed projections.

As described in TensoRF,^[^ ^60^ ^]^ a 3D volume (V) can be decomposed as a sum of the outer products of vector‐matrices:

(1) $$V=\sum_{r=1}^{R_{1}}M_{r}^{x,y}⊗v_{r}^{z}⊗v_{r}^{1}+\sum_{r=1}^{R_{2}}M_{r}^{x,z}⊗v_{r}^{y}⊗v_{r}^{2}+\sum_{r=1}^{R_{3}}M_{r}^{y,z}⊗v_{r}^{x}⊗v_{r}^{3}\;$$

where ⊗ is the outer product, $M_{r}^{x,y}⊗v_{r}^{z}⊗v_{r}^{1}$ is the component corresponding to different axes; $M_{r}^{x,y}\in R^{XY}$, $M_{r}^{x,z}\in R^{XZ}$, and $M_{r}^{z,y}\in R^{ZY}$ are matrices spanning the (*X*, *Y*), (*X*, *Z*), (*Y*, *Z*) axes and $V_{r}^{x}\in R^{X}$, $V_{r}^{y}\in R^{Y}$, $V_{r}^{z}\in R^{Z}$, and $V_{r}^{i}\in R^{F}$ are vectors of features. *R*_1_, *R*_2_, *R*_3_ are the number of low‐rank components. Factorization was known to reduce memory usage; however, factoring an independent 3D volume for each time step poses challenges due to sparse observations in the experimental configuration and the inability to share information across time points. To address this issue, the approach^[^ ^57^ ^]^ represented the 3D volume *V*_*t*_ as the weighted sum of a set of shared 3D basis volumes ${\hat{V}}_{i}$.

(2) $$V_{t}=\sum_{i=1}^{R_{t}}f{\left(t\right)}_{i}\cdot \hat{V_{i}}$$

Replacing $v_{r}^{1}\cdot f^{1}{\left(t\right)}_{r}$ with a joint function of *z* and *t*, similar to the matrix spanning the *X* and *Y* axes, Equation (1) becomes:

(3) $$\begin{array}{c} V_{4D}=\sum_{r=1}^{R_{1}}M_{r}^{x,y}⊗M_{r}^{z,t}⊗v_{r}^{1}+\sum_{r=1}^{R_{2}}M_{r}^{x,z}⊗M_{r}^{y,t}⊗v_{r}^{2}+\sum_{r=1}^{R_{3}}M_{r}^{y,z}⊗M_{r}^{x,t}⊗v_{r}^{3}\; \end{array}$$

where *V*_4*D*_ is the feature representation of a 4D volume, and each $M_{r}^{a,b}\in R^{A,B}$ is a learned plane of features, $v_{r}^{i}$ are the vectors of features. In this case, the spacetime complexity is reduced from *O*(*N^3^T*) to *O*(*N^2^F*), where *N*, *T*, and *F* are the spatial resolution, the temporal resolution, and feature size, significantly reducing the memory footprint. X‐rays were traced from each pixel of the projections along the direction in which the projections were acquired. Spacetime points were randomly selected along the X‐rays and projected onto the six planes. Six corresponding tensors were then extracted using bilinear interpolation. Specifically, each feature plane provides a *n*‐dimensional latent vector corresponding to its coordinate pair. These six feature vectors were concatenated to form a 6*n*‐D feature descriptor that compactly encodes both spatial and temporal characteristics of the queried point. This descriptor was then passed through an MLP, which regresses the complex index of refraction value *n*(**x**, *t*) at that specific coordinate.

After calculating *n*(**x**, *t*), 2D images or projections can be rendered using the projection approximation. The projection approximation can be described by:

(4) $$\psi_{z_{exit}}=\psi_{z_{0}}exp\left(-ik\int_{z_{0}}^{z_{exit}}\left(\delta \left[x,y;z\right]-i\beta \left[x,y;z\right]\right)dz\right)\;$$

where $\psi_{z_{0}}$ is the incoming wave on the sample, *k* = 2π/λ is the wavenumber which is inversely proportional to the wavelength (λ), δ, β are a function of the transverse coordinates *x* and *y* orthogonal to the propagation direction *z*. This formulation enabled the linear integration along the X‐ray propagation direction. Since the entire process was supervised through a physics‐based X‐ray forward model, the fused feature vector was trained to preserve physical consistency and faithfully represent the dynamic behavior of the samples.

X‐Hexplane was optimized by MSE loss between the rendered and target images. To address the ill‐defined sparse view problem, Total Variation and L1 loss were applied to six planes to enforce spatial‐temporal sparsity and continuity as regularizers. The optimization cost function or loss is given by:

(5) $$L=\frac{1}{\left|R\right|}\sum_{r\in R}{∥I\left(r\right)-\hat{I}\left(r\right)∥}_{2}^{2}+\lambda_{\text{reg}}L_{\text{reg}}$$

where *R* is the set of all points and $\hat{I}\left(r\right)$ denotes the estimated phase or attenuation contrast; *L*_*reg*_, λ_*reg*_ are regularization and its weight. By minimizing the difference between the real and predicted projections, the network parameters were updated, allowing it to produce more accurate and realistic 4D reconstructions.

In the presented studies, STRT was tailored to generate a single channel of the index of refraction. However, its adaptability and flexibility make it applicable to a wide range of time‐resolved imaging experiments across various imaging domains. For instance, it could be extended to techniques such as coherent diffraction imaging^[^ ^81^, ^82^ ^]^ and phase‐contrast imaging,^[^ ^62^, ^83^ ^]^ where the propagation model was explicitly known and could be easily incorporated into the X‐ray imaging model of XMPI.^[^ ^33^, ^48^ ^]^

### Network and Training Details

For the implementation, grid sizes for the six planes were adopted to scale according to the dimensions of the input dataset. In the drop collision case, the grid size for both spatial and temporal dimensions was set to 64. For the additive manufacturing case, the grid size was increased to 128 to accommodate the higher complexity of the scenario. In both cases, the embedded low‐rank tensors had a dimension of 48 for all grids, and the MLP architecture consisted of three layers, with each layer containing 64 neurons. This configuration ensured a balance between computational efficiency and model performance.

STRT was implemented using PyTorch 1.6.0 and Python 3.8.8. The optimization and training processes were conducted on an NVIDIA A100 GPU with 80 GB of RAM. The training time for 50,000 epochs was approximately 10 min for the droplet case and 60 min for the additive manufacturing case. The 3D rendering process required around 0.1 s per time point.

### Dataset Processing for Additive Manufacturing

Standard tomoscopy experiments for additive manufacturing were conducted in a continuous rotation mode while remelting structures that were produced with laser powder bed fusion. For each time point, projections within 0–180°were captured under the assumption that the sample did not change during this acquisition. The raw additive manufacturing dataset, initially sized (400, 200, 70, 528), representing 400 time points and 200 projections over 0–180°, and each with 70x528 pixels, was reshaped to (200, 400, 70, 528) for data extraction over 0–360°, resulting in half of the time points and twice as many projections. A flat‐field correction was applied to reduce background noise. This was followed by phase reconstruction using the method proposed by Paganin et al.^[^ ^73^ ^]^ to extract the phase information from edge enhancement. To further ensure consistency across projections, the Radon transform property was leveraged, which states that the sum of pixel values in each projection remains constant for each time point. This approach helps maintain data integrity and improves the accuracy of subsequent reconstructions.

Reconstructions using the conventional tomoscopy scheme with a full 0‐180°projection range, processed via the Gridrec algorithm,^[^ ^84^ ^]^ were used as ground truth to evaluate the method. The printing process of the additive manufacturing dataset occurred within 40 time points out of a total of 200 time points, therefore, only the printing period was evaluated.

## Conflict of Interest

The authors declare no conflict of interest.

## Author Contributions

Z.H. and P.V.‐P. conceived and conceptualized STRT. Z.H., Z. Y., Y.Z., and P.V‐P. developed and contributed to the neural network framework and physical formulation of the problem. Z.H. and K.J. performed the data analysis. M.M. and F. G.‐M. provided data and helped to devise experimental scenarios enabled by STRT. M.M. also contributed to the detailed analysis of the additive manufacturing results. P.V.‐P. supervised the research. Z. H. and P. V.‐P. wrote the article with input from all the coauthors.

## Supporting information

Supplemental Video 1

Supplemental Video 2

## Data Availability

The water droplet collision data that support the findings of this study are available in Figshare with the doi: https://doi.org/10.6084/m9.figshare.28533098.^[^ ^85^ ^]^ The code is available at https://github.com/huzhe8023‐a11y/X‐Hexplane.The additive manufacturing data that support the findings of this study are available in the PSI Public Data Repository with the doi: https://doi.org/10.16907/d64d2e8c‐b593‐47b8‐ab90‐4ddbd19bedb5.^[^ ^86^ ^]^
